# Stereotactic body radiotherapy for osseous low alpha–beta resistant metastases for pain relief—SOLAR-P

**DOI:** 10.1186/s13014-021-01897-0

**Published:** 2021-09-03

**Authors:** Eric K. Nguyen, Kimmen Quan, Sameer Parpia, Stephan Tran, Anand Swaminath

**Affiliations:** Department of Oncology, Juravinski Cancer Centre, McMaster University, 699 Concession Street, Hamilton, ON L8V 5C2 Canada

**Keywords:** SBRT, Stereotactic, Radiation, Pain, Bone, Metastases, Radio-resistant, Palliative

## Abstract

**Background:**

Stereotactic Body Radiotherapy (SBRT) has shown effectiveness in treating bone metastases to alleviate pain. The benefit of SBRT may be further harnessed especially when radiating disease from primary malignancies with low alpha–beta ratios in order to maximize the magnitude and durability of pain relief. However, such an approach has not been studied in a prospective trial. We look to assess single-fraction SBRT for painful non-spinal bone metastases from radioresistant primaries.

**Methods:**

Forty patients will be enrolled on an open label, phase II single arm trial to receive a single fraction of SBRT (15–20 Gray) to all sites of bone metastases requiring treatment for pain relief. Eligible patients will include those with primary malignancies consisting of prostate cancer, breast cancer, renal cell carcinoma, or melanoma. The primary endpoint is pain response at 3 months post-treatment using the Brief Pain Inventory. Secondary endpoints include pain response at 1 month and 6 months post-treatment, toxicity, patient-reported quality of life, re-irradiation or salvage surgery, and local control.

**Discussion:**

This study will evaluate the efficacy of single-fraction SBRT on painful bone metastases from primary cancers with low alpha–beta ratios. These data will be valuable to promote future randomized trials and support clinical implementation.

*Trial registration* Clinicaltrials.gov, NCT04177056. Date of registration: November 26, 2019. https://clinicaltrials.gov/ct2/show/NCT04177056

**Supplementary Information:**

The online version contains supplementary material available at 10.1186/s13014-021-01897-0.

## Background

The use of stereotactic body radiotherapy (SBRT) has been a topic of interest in palliative approaches for managing metastatic disease. Advances in radiation planning and delivery allows clinicians to target lesions with higher, ablative doses to tumors, while minimizing dose to organs and tissues at risk. SBRT has been demonstrated to be effective in controlling local metastatic lesions, while delaying distant progression of disease within the context of several retrospective, and small prospective randomized trials [1, 2].

The use of SBRT is appealing in particular for tumors with low alpha–beta ratios such as prostate cancer, breast cancer, renal cell carcinoma (RCC), and melanoma. Such tumors have inherent radioresistance to standard fractionation regimens, and benefit from dose escalation using hypofractionation to yield more potent biological efficacy [3, 4]. Theoretically, this may lead to amplification of vascular damage, increased endothelial cell apoptosis, and enhanced antitumor immunity [5]. Low alpha–beta tumors classically respond to higher doses per fraction, as demonstrated in pre-clinical studies for instance using RCC cell lines [6], and in prospective randomized trials suggesting equivalent or improved local control using hypofractionated radiation regimens for prostate cancer, breast cancer, and oligometastatic RCC [7–10]. The higher doses in fewer fractions theoretically may overcome innate tumor radioresistance resulting in improved local control and symptom resolution.

A recent phase II randomized trial included patients with bone metastases from all disease sites, and showed that single-fraction SBRT had higher rates of pain response at 2 weeks and 3 months compared to conventional fractionation [11]. However, the degree of pain response in the control group was less than expected and the majority of the patients had high alpha–beta tumors, most being lung cancer primaries. Furthermore, the radiation dose schedule given in the control arm was 30 Gy in 10 fractions which is less frequently used in the upfront treatment for uncomplicated bone metastases given its equivalence to shorter conventional treatment courses [12, 13].

Currently there is a paucity of prospective data studying the use of SBRT for bone metastases originating from low alpha–beta tumors, with systematic reporting of changes in pain scores and analgesia use over time. Most of the data regarding SBRT for bone lesions focuses on local control and survival, rather than more tangible outcomes in a palliative population including symptom control, durability of response (and need for retreatment), as well as patient reported quality of life; a component that is understudied in this group despite its tremendous value. Furthermore, SBRT for bone metastases has yet to become common practice given the limited evidence for its efficacy, relative complexity as compared to simple single- or multi-fraction palliative approaches, and uncertainty in regards to toxicity.

We propose an investigation of the potential benefits of SBRT for symptomatic bone metastases in patients with prostate cancer, breast cancer, RCC, and melanoma. We look to conduct a phase II single arm study (SOLAR-P) to assess pain response using this technique. We will also assess the tolerability of this modality, toxicity rates, and effect on quality of life.

## Methods

### Study design and patient population

This is an open label, phase II single-arm trial. Patients will be accrued from a single tertiary Canadian cancer centre. Eligibility criteria are listed below, and informed consent will be obtained for patients meeting all criteria.

### Inclusion criteria

- Diagnosis of prostate cancer, breast cancer, RCC, or melanoma
- Radiographic evidence of bone metastases requiring treatment for pain
- Brief Pain Inventory (BPI) score of ≥ 2 at baseline assessment

### Exclusion criteria

- Spinal lesions
- Impending (Mirels’ score ≥ 9) or existing pathological fracture
- Bone metastasis in a previously irradiated site
- Life expectancy < 3 months
- Age < 18
- Karnofsky Performance Status < 50
- Unable to provide informed consent
- Pregnant or breast-feeding women

### Primary outcome

- *Complete or partial pain response at 3 months—*Assessed using the BPI (online Appendix A) and converting daily analgesic use to oral morphine equivalent (OME)
  - *Complete response* defined as BPI pain score of 0 with no increase in OME.
  - *Partial response* defined as BPI pain score of > 0, and either a reduction of 2 or more with no increase in OME, or no increase in BPI with a reduction in OME or at least 25%.
  - *Treatment failure* defined as worsening pain on BPI by 2 or more, > 50% increase in OME, re-irradiation for pain/progression, or development of pathologic fracture.

### Secondary outcomes

- *Complete or partial pain response at 1 month and 6 months post SBRT—*Assessed using the BPI and response as described above.
- *Toxicity—*Acute (3 months or less), and late (greater than 3 months) adverse effects from RT will be recorded according to the Common Terminology Criteria for Adverse Events (CTCAE) 5.0 [17].
- *Patient-reported quality of life—*Quality of life assessed by European Organization of Research and Treatment of Cancer Quality of Life Questionnaire-Core-15-Palliative (EORTC QLQ-C15-PAL) and EORTC QLQ-Bone Metastasis 22 (EORTC QLQ-BM22), measured at 1 month, 3 month, and 6 months post SBRT [18, 19].
- *Re-irradiation or salvage surgery due to symptomatic progression—*Patients requiring re-irradiation will not undergo treatment for at least 4 weeks following the study radiation course. Patients requiring salvage surgery for disease progression, instability, or pathologic fractures will be reported.
- *Local control of treated lesions—*Defined as time of trial enrollment to date of radiographic progression. Radiographic control evaluated based on Response Evaluation Criteria in Solid Tumors (RECIST) 1.1 [20]. To be assessed retrospectively using standard of care follow-up imaging.

## Intervention and evaluation

### Pre-treatment evaluation

Patient eligibility will be determined during the initial assessment which includes a physical examination and review of radiographic evidence of metastases. Fracture risk will be assessed based on Mirels’ Staging System, taking into account the clinical evaluation and imaging available. For patients with high clinical suspicion of impending fracture, further evaluation and assessment may be taken as per standard of care.

An initial BPI will be completed prior to treatment during a clinic visit, or over the phone. Use of analgesic medications will be recorded.

### Intervention

**Fig. 1:**
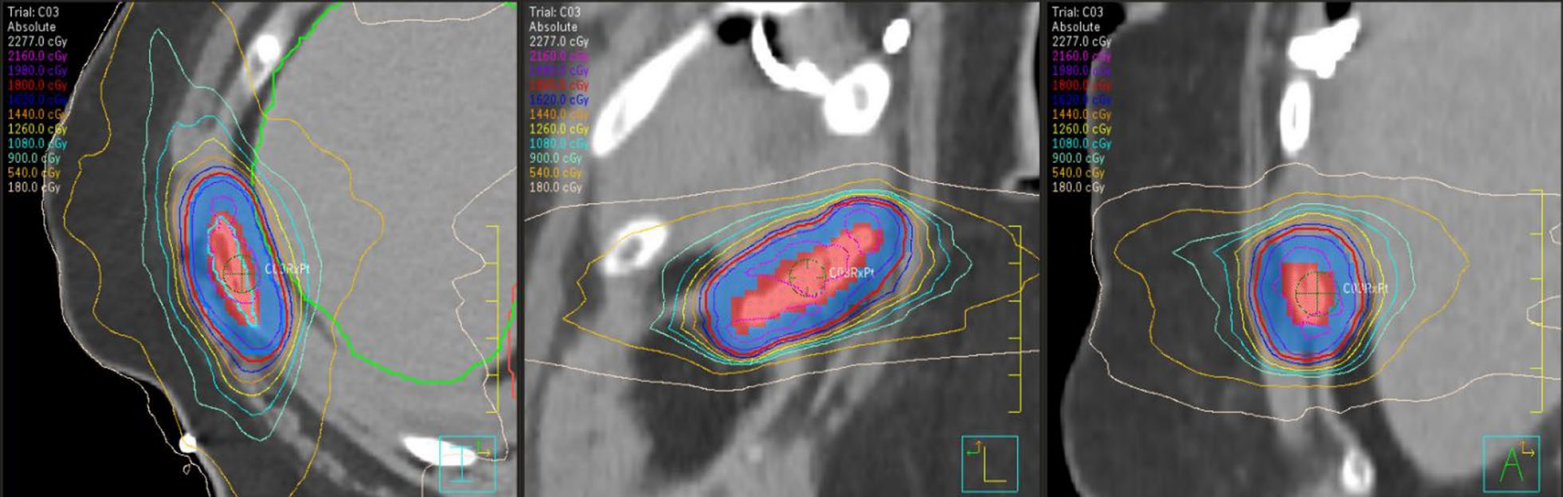
Treatment plan for a patient treated with 18 Gy in 1 fraction to the right rib

**Fig. 2:**
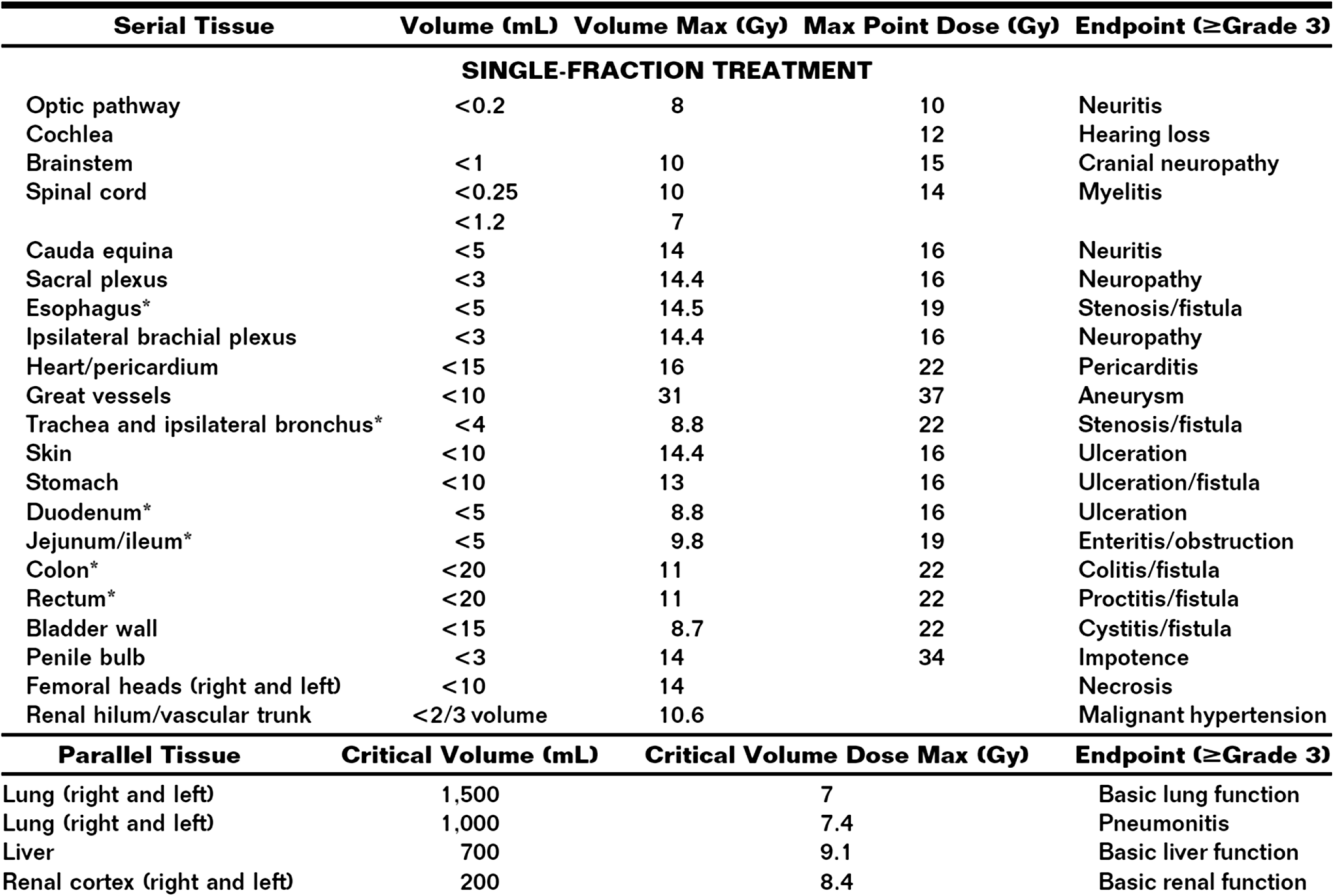
Organ at risk constraints [23]

Enrolled patients will receive the study dose of 15–20 Gy in 1 fraction to all painful bony lesions with SBRT. A dose range is given to facilitate safe treatment to larger lesions and metastases in close proximity to critical structures. If patients are on active systemic therapy, they will be taken off 5 days prior to, and after completion of SBRT. Planning and delivery will be conducted using a volumetric modulated arc therapy (VMAT) approach on the Varian Truebeam platform (Varian Medical Systems, Inc., Palo Alto, CA). Patients will be CT simulated, with custom vacuum-sealed cushions for immobilization, and MRI simulation may be performed. Use of 4DCT will be dependent on the area being treated but is not necessary for every case. The gross tumor volume (GTV) will be defined as the visible abnormality based on CT and MRI simulation imaging. A clinical target volume (CTV) may be contoured based on the characteristics of the target lesion and clinician preference. The planning target volume (PTV) will be an additional 5 mm in all directions. The SBRT prescription will ensure that at least 95% of the PTV will be covered with the prescribed dose, and that at least 99% of the PTV will be covered by 95% of the prescription dose (Fig. 1). Dose to organs at risk will based on published guidelines for single-fraction SBRT (Fig. 2). Daily image guidance will be performed using cone beam CT aligning to relevant bony anatomy ± PTV if visible. Priority will be made for patients to be seen, planned, and treated within 5 business days, akin to the usual standard of care for uncomplicated bone metastases. Peer review of all cases will take place prior to the start of treatment.

### Evaluation during the study

The primary outcome will be overall pain response, measured using the BPI. Patients will be assessed at baseline, and then at 1 month, 3 months, and 6 months following the completion of the SBRT treatment course. Responses will be obtained by patient self-reported questionnaires in clinic or by telephone follow-up. The sum of responses will dictate the overall response to treatment. Patients will be seen once for treatment review during the SBRT course to document acute toxicity. They will also be assessed at the 1 month, 3 month, and 6 month intervals to record acute and late toxicity using the CTCAE version 5.0. Quality of life will be measured using the EORTC QLQ-C15-PAL and BM22 questionnaires at the same timepoints.

### Subject discontinuation/withdrawal

Patients may discontinue participation in the study at any time, either prior treatment or decline follow up evaluations as per study protocol. In the case of an adverse event requiring study removal, the patient should have appropriate follow up by the treating physician for as long as necessary.

### Ethics

The study protocol and informed consent form have been reviewed and approved by the institutional ethics board prior to use in the trial. Appropriate ethics approval will be renewed yearly as per institutional standard.

## Sample size and statistical analysis

### Sample size

The estimated pain response (complete or partial) at 3 months using cRT is approximately 60%. We assume that under the null hypothesis, SBRT will have a similar response rate. We postulate that the pain response at 3 months using SBRT will be 80% (alternative hypothesis). Assuming 80% power, two-sided alpha of 0.05, we will require 40 patients to test this hypothesis using a one sample binomial test.

### Statistical analysis

The observed proportion of pain response at 3 months with SBRT will be compared to the null value of 0.6 using a one sample binomial test. The corresponding 95% confidence interval (CI) around the observed proportion will be calculated using the Wilson score method. Toxicity and quality of life will be summarized descriptively at each time point with corresponding CIs. Proportion of patient with who are re-irradiation or have salvage surgery due to symptomatic progression will be described with corresponding 95% CIs. Local control at will be estimated using the Kaplan–Meier method.

## Discussion

Conventional radiotherapy has been well established as an effective method in treating painful bone metastases, with 60% overall response rate for both single- and multi-fraction regimens [12, 14]. However, with the advent of targeted therapy and immuno-oncology, patients with metastatic disease are demonstrating improved systemic control and longer survival. Significant advancements in targeted therapy have extended outcomes in prostate cancer, breast cancer, RCC, and melanoma, and with this, durable pain control has become increasingly important for preserving quality of life [15–18]. With conventional fractionation, up to 20% of patients require retreatment and while re-irradiation is feasible, it can be complex to deliver safely with unknown response rates in low alpha–beta tumors [19]. Furthermore, with traditional fractionation, complete response rate is relatively low with only 23–24% of patients achieving this in prior studies [12].

SBRT has demonstrated promising results in the management of bone metastases. It has been shown to improve local control when treating bone lesions from prostate cancer with reductions of in-field failure in comparison to traditional schedules, but it is unclear whether this translates into long term pain relief [20]. Also, there has been early data in the use of SBRT for RCC bone metastases, given its resistance to conventional fractionation. One study showed that SBRT improved symptom control rates at 10, 12 and 24 months in comparison to standard RT, with a median time to control of 2 weeks, but documentation of pain control was variably captured and largely extrapolated from clinical notes [21].

Recently, results were presented for the Canadian Cancer Trials Group SC24 study which was a phase II/III trial examining the use of SBRT for spinal bone metastases for pain relief [22]. Patients with painful spine metastases were randomized to either SBRT with 24 Gy in 2 fractions, or conventional radiotherapy with 20 Gy in 5 fractions. The study did include breast cancer patients but excluded RCC primaries. At 3 months post-treatment, 35% of patients in the SBRT arm reported complete pain response from spinal lesions, compared to 14% in the conventional fractionated arm. There was no significant difference in adverse events between the two arms. This study supports the use of SBRT for spinal bone metastases with the primary purpose of pain reduction and highlights the advantages of high dose per fraction radiation in this patient population.

For the present study, we look to answer this question with respect to lesions from low alpha–beta primaries, excluding spinal lesions given the convincing evidence from SC24. A single-fraction regimen was chosen for the protocol to minimize treatment time while potentially giving superior symptomatic control from a radiobiological perspective. This allows for not only improved patient convenience, but also shorter breaks off systemic therapy. Furthermore, single-fraction SBRT may be more appropriate for smaller centers that do not have the resources to provide multi-fraction regimens. In summary, SOLAR-P is assessing the efficacy of single-fraction SBRT in the treatment of non-spinal bone metastases from low alpha–beta tumors for pain relief. The study looks to take advantage of the radiobiological difference in high dose radiotherapy in order to overcome radioresistance, and subsequently maximize symptom control.

## Supplementary Information

**Additional file 1.** Brief Pain Inventory.
