# Supplementary material for: Stereotactic body radiotherapy for osseous low alpha–beta resistant metastases for pain relief—SOLAR-P
Source: Radiat Oncol. 2021 Sep 3;16:170. doi: 10.1186/s13014-021-01897-0 (PMC8417953; doi:10.1186/s13014-021-01897-0)
Supplement: Supplementary file 1 — Additional file 1. Brief Pain Inventory. [file 13014_2021_1897_MOESM1_ESM.pdf]

1903

PLEASE USE BLACK INK PEN

Date: ____ / ____ / ____
(month) (day) (year)

Subject's Initials : ____________

Study Subject #: ____

Study Name: ______________________________

Protocol #: ______________________________

PI: ______________________________

Revision: 07/01/05

Brief Pain Inventory (Short Form)

1. Throughout our lives, most of us have had pain from time to time (such as minor headaches, sprains, and toothaches). Have you had pain other than these everyday kinds of pain today?

☐ Yes ☐ No

2. On the diagram, shade in the areas where you feel pain. Put an X on the area that hurts the most.

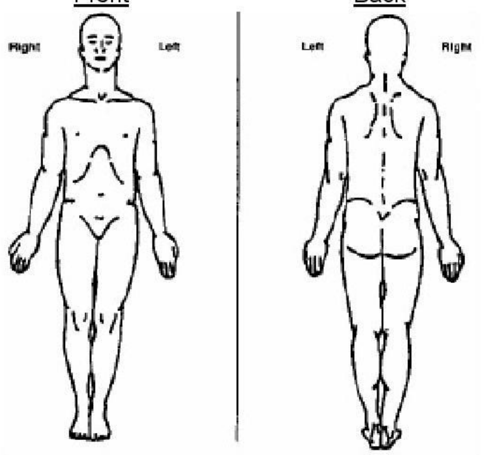

3. Please rate your pain by marking the box beside the number that best describes your pain at its worst in the last 24 hours.

☐ 0 ☐ 1 ☐ 2 ☐ 3 ☐ 4 ☐ 5 ☐ 6 ☐ 7 ☐ 8 ☐ 9 ☐ 10

No Pain — Pain As Bad As You Can Imagine

4. Please rate your pain by marking the box beside the number that best describes your pain at its least in the last 24 hours.

☐ 0 ☐ 1 ☐ 2 ☐ 3 ☐ 4 ☐ 5 ☐ 6 ☐ 7 ☐ 8 ☐ 9 ☐ 10

No Pain — Pain As Bad As You Can Imagine

5. Please rate your pain by marking the box beside the number that best describes your pain on the average.

☐ 0 ☐ 1 ☐ 2 ☐ 3 ☐ 4 ☐ 5 ☐ 6 ☐ 7 ☐ 8 ☐ 9 ☐ 10

No Pain — Pain As Bad As You Can Imagine

6. Please rate your pain by marking the box beside the number that tells how much pain you have right now.

☐ 0 ☐ 1 ☐ 2 ☐ 3 ☐ 4 ☐ 5 ☐ 6 ☐ 7 ☐ 8 ☐ 9 ☐ 10

No Pain — Pain As Bad As You Can Imagine

Copyright 1991 Charles S. Cleeland, PhD
Pain Research Group
All rights reserved

1903

PLEASE USE BLACK INK PEN

Date: ☐☐ / ☐☐ / ☐☐
(month) (day) (year)

Subject's Initials : ____________

Study Subject #: ☐☐☐☐

Study Name: ______________________________

Protocol #: ______________________________

PI: ______________________________

Revision: 07/01/05

7. What treatments or medications are you receiving for your pain?

8. In the last 24 hours, how much relief have pain treatments or medications provided? Please mark the box below the percentage that most shows how much relief you have received.

0%	10%	20%	30%	40%	50%	60%	70%	80%	90%	100%
☐ No Relief	☐	☐	☐	☐	☐	☐	☐	☐	☐	☐ Complete Relief

9. Mark the box beside the number that describes how, during the past 24 hours, pain has interfered with your:

A. General Activity

☐ 0 Does Not Interfere ☐ 1 ☐ 2 ☐ 3 ☐ 4 ☐ 5 ☐ 6 ☐ 7 ☐ 8 ☐ 9 ☐ 10 Completely Interferes

B. Mood

☐ 0 Does Not Interfere ☐ 1 ☐ 2 ☐ 3 ☐ 4 ☐ 5 ☐ 6 ☐ 7 ☐ 8 ☐ 9 ☐ 10 Completely Interferes

C. Walking ability

☐ 0 Does Not Interfere ☐ 1 ☐ 2 ☐ 3 ☐ 4 ☐ 5 ☐ 6 ☐ 7 ☐ 8 ☐ 9 ☐ 10 Completely Interferes

D. Normal Work (includes both work outside the home and housework)

☐ 0 Does Not Interfere ☐ 1 ☐ 2 ☐ 3 ☐ 4 ☐ 5 ☐ 6 ☐ 7 ☐ 8 ☐ 9 ☐ 10 Completely Interferes

E. Relations with other people

☐ 0 Does Not Interfere ☐ 1 ☐ 2 ☐ 3 ☐ 4 ☐ 5 ☐ 6 ☐ 7 ☐ 8 ☐ 9 ☐ 10 Completely Interferes

F. Sleep

☐ 0 Does Not Interfere ☐ 1 ☐ 2 ☐ 3 ☐ 4 ☐ 5 ☐ 6 ☐ 7 ☐ 8 ☐ 9 ☐ 10 Completely Interferes

G. Enjoyment of life

☐ 0 Does Not Interfere ☐ 1 ☐ 2 ☐ 3 ☐ 4 ☐ 5 ☐ 6 ☐ 7 ☐ 8 ☐ 9 ☐ 10 Completely Interferes

Copyright 1991 Charles S. Cleeland, PhD
Pain Research Group
All rights reserved
